# Assessing the Risk of Normal Weight Obesity in Korean Women across Generations: A Study on Body Composition and Physical Fitness

**DOI:** 10.3390/healthcare12111142

**Published:** 2024-06-04

**Authors:** Yeong-Hyun Cho, Hyuk Sakong, Myung-Jin Oh, Tae-Beom Seo

**Affiliations:** 1 Department of Sport Science, College of Natural Science, Jeju National University, 102 Jejudaehak-ro, Jeju 63243, Republic of Korea; choyh6839@jejunu.ac.kr; 2 Korea Institute of Sport Science, 727 Hwarang-ro, Nowongu, Seoul 01794, Republic of Korea; skhyuk9@gmail.com; 3 Division of Sports Science, Baekseok University, 1 Baekseokdaehak-ro, Dongnam-gu, Cheonan-si 31065, Republic of Korea; mjoh@bu.ac.kr

**Keywords:** normal weight obesity, across generations, Korean women, body composition, physical fitness

## Abstract

Normal weight obesity (NWO) refers to a condition in which the body mass index falls within the normal range, but the percent of body fat is excessive. Although there are reports of a high prevalence of cardiovascular and metabolic diseases in NWO, analyses regarding physical fitness have been lacking. Therefore, the purpose of this study was to analyze the age-related prevalence of NWO and to examine physical fitness across generations. Our study utilized a dataset comprising 119,835 participants for analysis. The prevalence of NWO across ages was examined using cross-tabulation analysis. For body composition and physical fitness, medians and group differences were assessed by generation through Kruskal–Wallis and Bonferroni post hoc tests. Additionally, univariate logistic regression was adopted to analyze the odds ratio. The prevalence of NWO in Korean women was 18.3%. The fat-free mass of the NWO group was consistently lower than that of both the group with normal body mass indexes (Normal) and obese body mass indexes (Obesity) across all generations. Additionally, the waist circumference and blood pressure were greater in the now group than in the Normal group. When considering maximal strength, muscle endurance, power, balance, and coordination, the NWO group exhibited lower levels compared to the Normal group. The NWO group showed lower muscle mass than both the Normal and Obesity groups, resulting in significantly reduced physical fitness compared to that of the Normal group, similar to the Obesity group. This condition may increase not only the risk of posing a potentially more serious health concern than obesity but also the risk of falls in elderly people. Therefore, based on this study, it is crucial to not only define obesity using BMI criteria but also to diagnose NWO. Public health policies and preventive measures must be implemented accordingly.

## 1. Introduction

The increasing prevalence of obesity in contemporary society is not only increasing the occurrence of hypertension, diabetes, cardiovascular disease, and nonalcoholic fatty liver but is also leading to a rising incidence of diverse diseases [1,2,3]. This condition, serving as a pivotal precursor to multiple health afflictions, is attributed to a multifaceted etiology encompassing genetic predisposition, socioeconomic influences, the heightened consumption of processed foods, and diminished levels of physical activity [4,5]. Consequently, addressing obesity and preventing related diseases are of paramount importance to improving readability [6].

The World Health Organization (WHO) has adopted BMI as a noninvasive and economical method because it considers only height and weight, and it has thus far used BMI to determine obesity [7]. However, despite these advantages, BMI has the disadvantage of not reflecting the percent of body fat [8].

Normal weight obesity (NWO), not classified as obesity due to BMI limitations, refers to individuals with a normal BMI but an excessive percentage of body fat [9]. This syndrome was first described by Ruderman et al. [10] as ‘Metabolically obese normal weight’ and was later named ‘Normal weight obesity’ by De Lorenzo et al. [11]. It is being studied as a new classification of metabolic disorders related to obesity [11,12].

NWO, characterized by a low skeletal muscle mass, has been reported to increase the risk of death from musculoskeletal diseases, cardiovascular disease, and metabolic syndrome [13,14,15]. In particular, studies have shown that women with NWO are 2.2 times more likely to die from cardiovascular disease than those with NWO due to hormonal effects [4,16,17]. Moreover, elderly individuals may be more vulnerable to musculoskeletal and cardiovascular diseases in NWO [18,19]. Furthermore, a combination of obesity and sarcopenia can accelerate elderly individuals’ symptoms, reducing their exercise capacity. Thorough management is needed to prevent NWO [20,21].

According to several studies, the worldwide prevalence of NWO is reported to be 9–34% [14,16,22], especially among Asians, who have a high prevalence of metabolic disorders despite having a lower weight than individuals from other continents [11,23,24]. This indicates that the prevalence and severity of obesity are underestimated due to the smaller physique of Asians. In Korea, there is also a potential hidden risk of NWO, underscoring the imperative need for dedicated research on NWO among Koreans.

Therefore, the purpose of this study was to investigate NWO among Korean women across different generations (Youth, Middle-aged, Elderly) and to analyze body composition and physical fitness to determine the associated risks.

## 2. Materials and Methods

### 2.1. Study Design

The data utilized in this investigation were acquired by the Korea Sports Promotion Foundation, covering the period from January 2019 to March 2023, and sourced from the Big Data Platform of the Korea Culture Information Service Agency (www.bigdata-culture.kr) (accessed on 1 June 2023). Stringent adherence to privacy guidelines was upheld, with only essential information relevant to the study being disclosed. Ethical approval for consent exemption was obtained from the Jeju National University Institutional Review Board (JJNU-IRB-2024-013). Out of the 565,463 provided data points, 119,835 were employed for the analysis of NWO prevalence by generation in adult women, excluding males, individuals under 19 years of age, those with unmeasured body components, and data entries deemed incorrect (n = 445,628).

In this study, the underweight population was excluded (n = 6975). During the body composition and physical fitness analytical phase for the normal, NWO, and obesity populations, only data with all physical fitness variables measured were utilized. Additionally, for the generational analysis, individuals aged 19 to 44 years were categorized as Youth (n = 27,257), those aged 45 to 64 years were categorized as Middle-aged (n = 17,588), and individuals aged 65 years and older were categorized as Elderly (n = 13,082).

### 2.2. Subjects

Our study aimed to analyze the prevalence of NWO across different generations (Youth, Middle-aged, and Elderly) and assess the associated body composition and physical fitness levels to determine the risk of NWO. The group composition was categorized as the group with underweight (Under), normal body mass indexes (Normal), Normal weight obesity (NWO), and obese body mass indexes (Obesity), based on the obesity judgment criteria established by the WHO in the Asia-Pacific Obesity Standard [25]. Accordingly, the standard for underweight individuals is a BMI < 18.5 kg/m^2^, the standard for overweight individuals is a BMI ≥ 23 kg/m^2^, and the standard for normal range individuals is 18.5 kg/m^2^ ≤ BMI < 23 kg/m^2^.

NWO is characterized by a population with a BMI within the normal range but with an excess body fat percentage. The cutoff determining excessive body fat rates in NWO varies across studies, with substantial variations observed by race and age region. The NWO criteria for this study are as follows; BMI 18.5 kg/m^2^ ≤ BMI < 23 kg/m^2^ were classified as normal, those with a BMI 18.5 kg/m^2^ ≤ BMI < 23 kg/m^2^ but with a body fat percentage over 30% were classified as having normal weight obesity, and those with a BMI ≥ 23 kg/m^2^ were classified as having obesity The characteristics of the study participants are shown in Table 1.

### 2.3. Measurement

#### 2.3.1. Anthropometric Measurements and Body Composition

Height was measured using a stadiometer (Seca Corporation, Columbia, MD, USA) with a precision of 0.1 cm, while weight and body composition were assessed using bioelectrical impedance analysis (Inbody 720, Biospace, Seoul, Republic of Korea) with a precision of 0.1 kg.

#### 2.3.2. Blood Pressure

For blood pressure measurements, upon arrival at the laboratory, the participants were allowed sufficient rest, a standard adult cuff was placed on their left upper arm, and blood pressure was measured using a blood pressure monitor (HEM-7121J, Omron, Kyoto, Japan). Measurements were conducted twice, and the minimum recorded values for both systolic blood pressure (SBP) and diastolic blood pressure (DBP) were utilized.

#### 2.3.3. Maximal Grip Strength Test

The maximal grip strength test (MGS), a measure of maximal strength, was evaluated using a hand dynamometer (Grip-D5101, Takei, Niigata, Japan). When measuring grip strength, the participants exerted maximum force on the dynamometer, with the angle between the arm and the torso maintained at 15°. The dynamometer handle was positioned between the second interphalangeal joint of the four fingers and the base of the thumb’s palm. MGS was measured twice for each hand, and the highest value in 0.1 kg units was recorded.

#### 2.3.4. Sit-Up Test

The sit-up test (SU) utilized a muscle endurance measuring device (BS-SU, Inbody, Seoul, Republic of Korea). The participants were instructed to lie supine, cross their hands over their chest, and position their knees at a 45-degree angle. Each time the participants touched their knee and returned to a supine position during the measurement, it was counted as one repetition. The maximum number of repetitions was measured for one minute. The elderly were not measured due to their risk of injury.

#### 2.3.5. Standing Long Jump Test

The standing long jump (SLJ) test was used to evaluate power. The participants were instructed to stand at the starting line and jump forward as much as possible. The measurements were taken from the starting line to the landing point of the nearest heel. The test was conducted on two occasions, and the maximum distance was recorded.

#### 2.3.6. 30 s Chair Stand Test

The 30 s chair stand test (30 s CST) was used to evaluate the muscular strength and endurance of the lower limbs in the elderly participants. The participants were seated centrally in a chair, with their hands crossed at opposite shoulders and wrists and feet positioned flat on the floor. Upon a signal, the participants were directed to perform full standing and sitting movements for 30 s. The maximum number of repetitions was recorded, and given the age of the participants, the test was promptly halted if they experienced any bodily discomfort.

#### 2.3.7. Timed Up and Go

The TUG (Timed Up and Go) test is a method for assessing the balance ability of elderly individuals; this test involves getting up from a 46 cm-tall chair without an armrest, circling a conical arrangement 3 m apart, returning to the starting point, and then sitting again. The measurement was recorded from the moment the hips of the participants started to be lifted out of the chair to the point at which the hips touched the seat. The TUG test was conducted twice in a row, and the average score from the two measurements in 0.1 s units was recorded.

#### 2.3.8. Figure-of-8 Walk

The figure-of-8 walk test (F8WT) was employed to assess the coordination, walking ability, and overall mobility of elderly individuals. Cones were placed at both vertices in the rectangular area (3.5 m × 1.6 m) of the floor. The lower midpoint of the rectangle was a chair situated 2.4 m from each cone. Upon the start signal, the participants sat on the chair, stood up, navigated around the cone at the rear right, returned to the chair, sat down, navigated around the cone on the left, returned to the chair, and sat down. The elapsed time for the entire task was measured twice using a stopwatch, and the fastest recorded value in 0.1 s units was recorded.

### 2.4. Statistical Analysis

**Figure 1:**
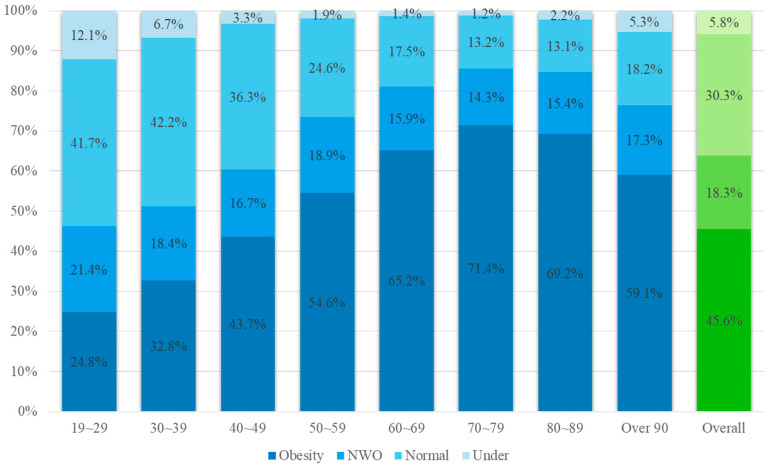
Demographic analysis on obesity levels according to ages; NWO, normal weight obesity (n = 119,835).

In our study, we extracted body composition data from adult women to analyze the prevalence of NWO by age. We classified individuals into 10-year age groups through cross-tabulation analysis, investigated the prevalence by age, expressed it as a percentage, and presented it in Figure 1. The subjects’ characteristics are presented as the mean ± standard deviation throughout the descriptive statistics. Independent variations in generational NWO were compared and analyzed, and as a result of verifying normality before comparison, a nonparametric Kruskal–Wallis test was performed because normality was not achieved. Medians along with the 25th–75th percentiles were calculated. Bonferroni post hoc analysis was employed when significant differences in body composition were observed. Logistic regression analysis was used to assess the influence of variables on outcomes, and in particular, univariate logistic regression analysis was adopted to analyze the effect of each independent variable on outcomes. Univariate logistic regression was performed with the reference variable set to the Normal group, and the odds ratios (ORs) and 95% confidence intervals (CIs) were calculated.

## 3. Results

### 3.1. Demographic Analysis of the Obesity Levels

Figure 1 presents an analysis of obesity levels among 119,835 adult women, showing that 45.6% of the total population were obese, 18.3% had NWO, 30.3% had a normal weight, and 5.8% were underweight. Additionally, to examine obesity levels by age, cross-tabulation analysis was conducted in 10-year increments, and the results are presented as percentages.

### 3.2. Comparative Analysis of Body Compositions, Blood Pressure, and Physical Fitness in Young Women

**Figure 2:**
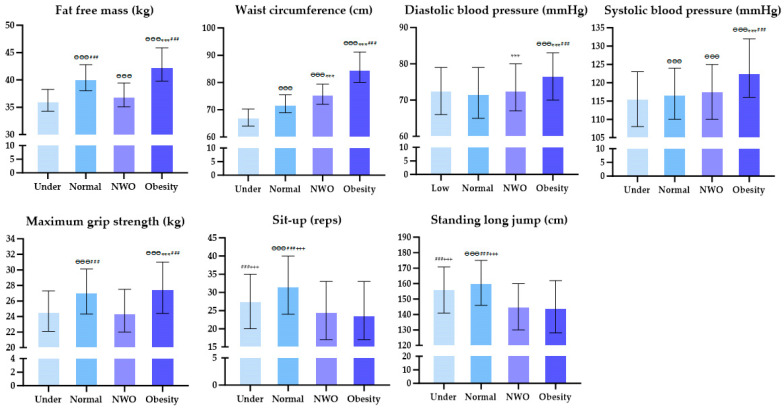
Visualization of body composition, blood pressure, and physical fitness in young women according to obesity level; NWO, normal weight obesity. ^ӨӨӨ^, *p* < 0.001 vs. Under; ***, *p* < 0.001 vs. Normal; ^###^ *p* < 0.001 vs. NWO; ^+++^ *p* < 0.001 vs. Obesity.

Figure 2 presents the results of analyzing the body composition, blood pressure, and physical fitness in youth according to obesity levels through Kruskal–Wallis analysis and Bonferroni post hoc tests. The analysis revealed significant differences in all variables, and the significant post hoc results are presented in the graphs. Table 2 and Table 3 present the results of the logistic regression, which was conducted to analyze the impact between groups. The analysis demonstrated that the NWO group had lower fat-free mass, MGS, SU, and SLJ values than the Normal group, while the waist circumference and diastolic blood pressure were higher. The Obesity group had lower SU and SLJ values than the Normal group and a greater waist circumference, DBP, SBP, and MGS. The underweight group had lower fat-free mass, WC, and SBP values than the Normal, now, and Obesity groups, while the SU and SLJ values were higher than those in the NWO group. The fat-free mass was 24% lower in the NWO group than in the Normal group (OR = 0.76, 95% CI 0.76–0.77, *p* = 0.001) and 31% lower in the Under group (OR = 0.69, 95% CI 0.68–0.70, *p* = 0.001). However, the Obesity group had a 16% higher fat-free mass (OR = 1.16, 95% CI 1.16–1.17, *p* = 0.001). The MGS was 12% lower in the NWO (OR = 0.88, 95% CI 0.87–0.89, *p* = 0.001) and Under groups (OR = 0.88, 95% CI 0.88–0.89, *p* = 0.001) than in the Normal group, while that in the Obesity group was 2% higher (OR = 1.02, 95% CI 1.01–1.03, *p* = 0.001).

### 3.3. Comparative Analysis of Body Compositions, Blood Pressure, and Physical Fitness in Middle-Aged Women

**Figure 3:**
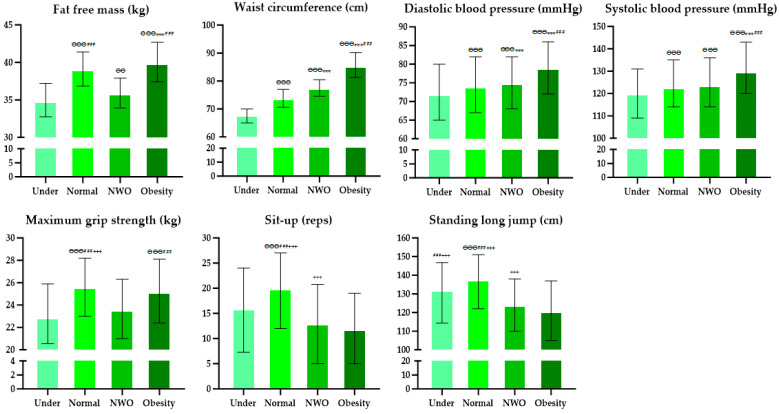
Visualization of body composition, blood pressure, and physical fitness in middle-aged women according to obesity level; NWO, normal weight obesity. ^ӨӨ^, *p* < 0.01 vs. Under; ^ӨӨӨ^, *p* < 0.001 vs. Under; ***, *p* < 0.001 vs. Normal; ^###^ *p* < 0.001 vs. NWO; ^+++^ *p* < 0.001 vs. Obesity.

Figure 3 displays the outcomes of examining the body composition, blood pressure, and physical fitness among middle-aged individuals, categorized by obesity level through Kruskal–Wallis analysis and Bonferroni post hoc tests. The analysis revealed significant differences across all variables, with noteworthy post hoc results visualized in the graphs. Table 4 and Table 5 show the findings from a logistic regression designed to explore the impact between groups. The analysis revealed that the NWO group exhibited lower fat-free mass, maximal grip strength, sit-up, and standing long jump values compared to the Normal group. Conversely, the WC, DBP, and SBP were higher. The Obesity group had lower MGS, SU, and SLJ values, as well as larger WC, DBP, and SBP values, than the Normal group. The Under group had lower fat-free mass, WC, DBP, and SBP values than the Normal, NWO, and Obesity groups, while their SU and SLJ values were higher than those of the NWO group. In particular, the fat-free mass was 25%~32% lower in the NWO (OR = 0.75, 95% CI 0.74–0.76, *p* = 0.001) and Under (OR = 0.68, 95% CI 0.65–0.70, *p* = 0.001) groups than in the Normal group, while the Obesity group presented a 7% higher fat-free mass (OR = 1.07, 95% CI 1.06–1.08, *p* = 0.001).

### 3.4. Comparative Analysis of Body Compositions, Blood Pressure, and Physical Fitness in Elderly Women

**Figure 4:**
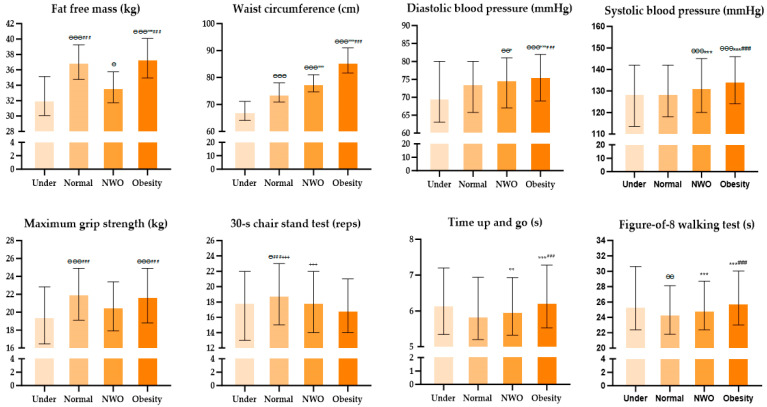
Visualization of body composition, blood pressure, and physical fitness in elderly women according to obesity level; NWO, normal weight obesity. ^Ө^, *p* < 0.05 vs. Under; ^ӨӨ^, *p* < 0.01 vs. Under; ^ӨӨӨ^, *p* < 0.001 vs. Under; *, *p* < 0.05 vs. Normal; **, *p* < 0.01 vs. Normal; ***, *p* < 0.001 vs. Normal; ^###^ *p* < 0.001 vs. NWO; ^+++^ *p* < 0.001 vs. Obesity.

Figure 4 shows the findings from an investigation into the body composition, blood pressure, and physical fitness among elderly individuals stratified by obesity level using Kruskal–Wallis analysis and Bonferroni post hoc tests. The analysis revealed notable differences across all variables, with significant post hoc results graphically presented. Table 6 and Table 7 provide insights from a logistic regression designed to explore the impact between groups. The analysis highlighted that the NWO group exhibited a lower fat-free mass, MGS, and 30 s CST than the Normal group. Conversely, the DBP, SBP, and waist circumference were higher. Compared with the Normal group, the Obesity group had a lower 30 s CST and a greater fat-free mass, waist circumference, DBP, SBP, TUG, and F8WT. The MGS was not significantly different from that of the Normal group. The Under group had a lower fat-free mass and WC than the Normal, now, and Obesity groups, while the F8WT was higher than that in the Normal group. Specifically, the fat-free mass was 23%~31% lower in the NWO (OR = 0.77, 95% CI 0.76–0.76, *p* = 0.001) and Under (OR = 0.69, 95% CI 0.67–0.72, *p* = 0.001) groups than in the Normal group, while that in the Obesity group was 3% higher (OR = 1.03, 95% CI 1.02–1.04, *p* = 0.001).

## 4. Discussion

Our study investigated the prevalence of NWO in 119,835 Korean adult women and analyzed the body composition, blood pressure, and physical fitness. The analysis revealed an 18.3% prevalence of NWO among Korean adult women, indicating an incidence rate that is not low compared to the global prevalence rate (ranging from 9% to 34%). This finding aligns with those of Kapoor et al. [11], who reported a higher prevalence of NWO in Asians, attributed to their relatively smaller body size compared to other ethnicities. Therefore, NWO syndrome should not be overlooked in Koreans.

To date, studies have consistently reported that individuals with NWO exhibit elevated levels of inflammatory and prothrombotic biomarkers, making them more susceptible to cardiovascular and metabolic diseases than those with a normal weight [26,27,28]. In our study, we investigated the waist circumference and blood pressure across different age groups and revealed that the NWO group had larger waist circumferences than the Normal group. Additionally, their diastolic blood pressure was found to be greater than that in the Normal group.

Similarly, previous studies have reported that individuals with NWO have a greater waist circumference and blood pressure, increasing their risk of diabetes, hypertension, and dyslipidemia [22,29]. Both waist circumference and blood pressure are crucial factors in diagnosing metabolic syndrome, and our findings, along with those of prior research, support the assertion that NWO contributes to a greater incidence of metabolic syndrome.

While most studies have focused primarily on elucidating the implications of NWO in relation to the risk of immune and hematological diseases [16], other associated risks should not be disregarded. A low muscle mass is closely correlated with musculoskeletal and cardiovascular diseases, as well as increased mortality, leading to diminished exercise capacity and an elevated risk of various injuries. This study revealed that the fat-free mass in the NWO group was 24% lower in youth, 25% lower in middle-aged individuals, and 23% lower in the elderly compared to the Normal group, and it was even lower than in the Obesity group.

It has been reported that postmenopausal hormonal changes in women inevitably result in increased body fat and reduced muscle mass [30], and obesity in women can amplify sarcopenia [31]. Obesity and low muscle mass in elderly individuals can hasten the onset of geriatric syndromes, contributing to fall accidents and worsening the quality of life for elderly individuals with weakened immunity and resilience. Our study revealed that the Obesity group had 3% more fat-free mass than the Normal group, whereas the NWO group had 23% less lean mass than the Normal group. These findings are particularly significant considering that muscle loss due to aging has already occurred to some extent in individuals with a normal weight. This suggests that muscle mass loss in NWO could be particularly detrimental. Individuals with NWO are considered to be the most vulnerable to various diseases caused by sarcopenia compared to both individuals with normal weight and individuals with obesity.

It has been reported that sarcopenia can diminish physical fitness and exercise capacity [31]. In this study, we conducted an analysis of health-related physical fitness, including maximal strength, muscular endurance, power, balance, and coordination. Maximal grip strength, a measurement tool for assessing partial maximal strength, has been shown in various studies to predict not only maximal strength but also conditions such as type 2 diabetes, coronary artery disease (CAD), metabolic syndrome, and mortality [32,33,34]. In our study, the NWO and Under groups exhibited significantly lower maximal strengths than both the Normal and Obesity groups. These findings may be attributed to a lower muscle mass, and it was observed that individuals with NWO had notably lower levels of muscle strength. Consequently, the reduced muscle strength in individuals with NWO may indicate a greater likelihood of cardiovascular and metabolic diseases.

Muscular endurance and power are crucial components of physical fitness that, along with muscle strength, determine exercise capacity. In this study, muscular endurance and power were assessed using SU and SLJ for youth and middle-aged individuals. Power was not measured for elderly people due to the risk of injury, and muscular endurance was assessed using the 30 s CST. The results of the measurements revealed that the NWO and Under groups exhibited lower levels of both muscular endurance and power than the Normal group, similar to the Obesity group. Both muscular endurance and power are physical fitness factors related to muscle strength, and these results are thought to be caused by the weakening of muscle strength [35].

Reportedly, more than 30% of falls in elderly individuals occur annually, with a 50% recurrence rate [36], and severe injuries such as fractures and brain damage are directly linked to mortality [37]. This study assessed the ability of balance and coordination in elderly people in predicting falls [38]. The findings revealed that the NWO and Under groups had significantly lower scores for balance and coordination than the Normal group. A previous study identified obesity in elderly people as a factor contributing to increased fall incidents, citing reduced muscle mass and compromised neuromuscular function as causal factors [39,40]. In this study, NWO was associated with both obesity and decreased muscle mass, suggesting that the risk of falling may be higher than that of obesity.

This study has several limitations. First, we were unable to evaluate other diseases, blood variables, or nutritional factors that could influence an individual’s physical fitness level. Future analyses correlating these factors with major diseases could reveal a direct relationship with physical fitness. The second limitation stems from the cross-sectional nature of the study, which prevents the follow-up of subjects to track changes over time. Third, the study included only data from women, so the results cannot be generalized to men. Despite these limitations, the study is notable for its large scale. We are confident in the originality of our work, as it is the first to analyze the relationship between NWO and physical fitness across different generations. In the future, national support for the development and distribution of specific exercise programs, along with education on the risks of NWO with adolescents, is essential to addressing the increasing concern regarding both obesity and NWO.

## 5. Conclusions

This study revealed an 18.3% prevalence of NWO among Korean adult women. Additionally, NWO is associated with a lower muscle mass compared to obesity, significantly reducing physical fitness. This suggests that individuals with NWO may have a higher incidence of various diseases and a greater risk of falling than those with obesity. The modernization and convenience of contemporary society have increased the prevalence of NWO among Korean women, negatively impacting all generations. Therefore, NWO should be classified as an even more dangerous syndrome than obesity. Based on this study, it is crucial to not only define obesity using BMI criteria but also diagnose NWO. Public health policies and preventive measures must be implemented accordingly.

## Figures and Tables

**Table 1:** Characteristics of Subjects by Generation.

Variable		Under (n = 3240)	Normal (n = 19,133)	NWO (n = 11,174)	Obesity (n = 27,620)
Youth	Height (cm)	163.36 ± 4.93	162.89 ± 5.10	161.73 ± 5.23	161.41 ± 5.48
	Weight (kg)	46.97 ± 3.40	54.08 ± 4.47	55.55 ± 4.51	68.76 ± 9.49
	%BF (%)	22.66 ± 3.81	25.05 ± 3.42	32.92 ± 2.32	36.97 ± 4.48
Middle-aged	Height (cm)	158.49 ± 5.65	158.52 ± 5.11	157.23 ± 5.05	156.51 ± 5.19
	Weight (kg)	44.36 ± 3.66	52.67 ± 4.37	53.79 ± 4.10	63.88 ± 7.35
	%BF (%)	21.31 ± 4.51	25.44 ± 3.42	33.22 ± 2.46	36.85 ± 4.08
Elderly	Height (cm)	153.89 ± 5.98	153.96 ± 5.68	152.82 ± 5.54	152.41 ± 5.57
	Weight (kg)	41.19 ± 4.14	49.83 ± 4.55	50.94 ± 4.21	61.05 ± 6.96
	%BF (%)	21.70 ± 6.12	25.40 ± 3.82	33.80 ± 2.84	38.22 ± 4.52

Values are presented as the mean ± standard deviation. NWO, normal weight obesity; %BF, percent body fat.

**Table 2:** Comparative analysis of body composition, blood pressure, and physical fitness in young women according to obesity level.

Variable	Under (n = 3240)	Normal (n = 12,661)	NWO (n = 6062)	Obesity (n = 8534)	*p*
FFM (kg)	36.23 (34.25–38.26)	40.31 ^ӨӨӨ###^ (38.03–42.77)	37.20 ^ӨӨӨ^ (35.06–39.42)	42.60 ^ӨӨӨ^***^###^ (39.78–45.88)	0.001
WC (cm)	67.70 (64.00–70.30)	72.20 ^ӨӨӨ^ (68.90–75.50)	76.00 ^ӨӨӨ^*** (72.00–79.40)	85.10 ^ӨӨӨ^***^###^ (80.00–91.20)	0.001
DBP (mmHg)	73.00 (66.00–79.00)	72.00 (65.00–79.00)	73.00 *** (67.00–80.00)	77.00 ^ӨӨӨ^***^###^ (70.00–83.00)	0.001
SBP (mmHg)	116.00 (108.00–123.00)	117.00 ^ӨӨӨ^ (110.00–124.00)	118.00 ^ӨӨӨ^ (110.00–125.00)	123.0 ^ӨӨӨ^***^###^ (116.00–132.00)	0.001
MGS (kg)	24.70 (22.10–27.30)	27.20 ^ӨӨӨ###^ (24.30–30.10)	24.60 (22.00–27.50)	27.70 ^ӨӨӨ^***^###^ (24.40–31.00)	0.001
SU (reps)	28.00 ^###+++^ (20.00–35.00)	32.00 ^ӨӨӨ###+++^ (24.00–40.00)	25.00 (17.00–33.00)	24.00 (17.00–33.00)	0.001
SLJ (cm)	157.00 ^###+++^ (141.00–170.75)	161.00 ^ӨӨӨ###+++^ (146.00–175.00)	146.00 (130.00–160.00)	145.00 (128.00–162.00)	0.001

NWO, Normal weight obesity; FFM, Fat-free mass; WC, Waist circumference; DBP, Diastolic blood pressure; SBP, Systolic blood pressure; MGS, Maximal grip strength; SU, Sit-up; SLJ, Standing long jump; ^ӨӨӨ^, *p* < 0.001 vs. Under; ***, *p* < 0.001 vs. Normal; ^###^ *p* < 0.001 vs. NWO; ^+++^ *p* < 0.001 vs. Obesity.

**Table 3:** The logistic regression analysis based on the obesity level with the reference variable ‘Normal’ in young women.

	Under		NWO		Obesity	
	OR (95% CI)	*p*	OR (95% CI)	*p*	OR (95% CI)	*p*
FFM (kg)	0.69 (0.68–0.70)	0.001	0.76 (0.76–0.77)	0.001	1.16 (1.16–1.17)	0.001
WC (cm)	0.84 (0.83–0.85)	0.001	1.13 (1.12–1.14)	0.001	1.43 (1.42–1.44)	0.001
DBP (mmHg)	1.00 (1.00–1.00)	0.821	1.01 (1.00–1.01)	0.001	1.05 (1.04–1.05)	0.001
SBP (mmHg)	0.99 (0.98–0.99)	0.001	1.00 (1.00–1.00)	0.659	1.04 (1.04–1.05)	0.001
MGS (kg)	0.88 (0.87–0.89)	0.001	0.88 (0.88–0.89)	0.001	1.02 (1.01–1.03)	0.001
SU (reps)	0.97 (0.97–0.98)	0.001	0.96 (0.95–0.96)	0.001	0.96 (0.95–0.96)	0.001
SLJ (cm)	0.99 (0.99–0.99)	0.001	0.97 (0.97–0.97)	0.001	0.97 (0.97–0.97)	0.001

NWO, Normal weight obesity; FFM, Fat-free mass; WC, Waist circumference; DBP, Diastolic blood pressure; SBP, Systolic blood pressure; MGS, Maximal grip strength; SU, Sit-up; SLJ, Standing long jump; CI, confidence interval.

**Table 4:** Comparative analysis of body composition, blood pressure, and physical fitness in middle-aged women according to obesity level.

Variable	Under (n = 344)	Normal (n = 4514)	NWO (n = 3180)	Obesity (n = 9894)	*p*
FFM (kg)	34.84 (32.75–37.20)	39.11 ^ӨӨӨ###^ (36.84–41.41)	35.91 ^ӨӨ^ (33.92–37.89)	39.92 ^ӨӨӨ^***^###^ (37.43–42.66)	0.001
WC (cm)	68.00 (65.00–70.00)	73.90 ^ӨӨӨ^ (70.60–77.00)	77.70 ^ӨӨӨ^*** (74.60–80.50)	85.40 ^ӨӨӨ^***^###^ (81.30–90.20)	0.001
DBP (mmHg)	72.00 (65.00–80.00)	74.00 ^ӨӨӨ^ (67.00–82.00)	75.00 ^ӨӨӨ^*** (68.00–82.00)	79.00 ^ӨӨӨ^***^###^ (72.00–86.00)	0.001
SBP (mmHg)	120.00 (109.00–131.00)	123.00 ^ӨӨӨ^ (114.00–135.00)	124.00 ^ӨӨӨ^ (114.00–136.00)	130.00 ^ӨӨӨ^***^###^ (120.00–143.00)	0.001
MGS (kg)	22.90 (20.53–25.88)	25.60 ^ӨӨӨ###+++^ (23.00–28.20)	23.60 (21.00–26.30)	25.20 ^ӨӨӨ###^ (22.40–28.10)	0.001
SU (reps)	16.00 (7.25–24.00)	20.00 ^ӨӨӨ###+++^ (12.00–27.00)	13.00 ^+++^ (5.00–20.75)	12.00 (5.00–19.00)	0.001
SLJ (cm)	132.50 ^###+++^ (114.25–146.75)	138.00 ^ӨӨӨ###+++^ (122.00–151.00)	124.00 ^+++^ (110.00–138.00)	121.00 (105.00–137.00)	0.001

Values are presented as the mean ± standard deviation. NWO, Normal weight obesity; FFM, Fat-free mass; WC, Waist circumference; DBP, Diastolic blood pressure; SBP, Systolic blood pressure; MGS, Maximal grip strength; SU, Sit-up; SLJ, Standing long jump; ^ӨӨ^, *p* < 0.01 vs. Under; ^ӨӨӨ^, *p* < 0.001 vs. Under; ***, *p* < 0.001 vs. Normal; ^###^ *p* < 0.001 vs. NWO; ^+++^ *p* < 0.001 vs. Obesity.

**Table 5:** Results of the odds ratio analysis of obesity regarding the body composition, blood pressure, and physical fitness of middle-aged women.

	Under		NWO		Obesity	
	OR (95% CI)	*p*	OR (95% CI)	*p*	OR (95% CI)	*p*
FFM (kg)	0.68 (0.65–0.70)	0.001	0.75 (0.74–0.76)	0.001	1.07 (1.06–1.08)	0.001
WC (cm)	0.78 (0.76–0.80)	0.001	1.16 (1.15–1.17)	0.001	1.51 (1.49–1.52)	0.001
DBP (mmHg)	0.98 (0.97–0.99)	0.001	1.01 (1.00–1.01)	0.001	1.04 (1.04–1.05)	0.001
SBP (mmHg)	0.98 (0.97–0.99)	0.001	1.00 (1.00–1.01)	0.014	1.03 (1.03–1.03)	0.001
MGS (kg)	0.87 (0.85–0.89)	0.001	0.90 (0.89–0.91)	0.001	0.98 (0.98–0.99)	0.001
SU (reps)	0.97 (0.96–0.98)	0.001	0.94 (0.94–0.94)	0.001	0.93 (0.93–0.93)	0.001
SLJ (cm)	0.99 (0.98–0.99)	0.001	0.97 (0.97–0.98)	0.001	0.97 (0.97–0.97)	0.001

Values are presented as the mean ± standard deviation. NWO, Normal weight obesity; FFM, Fat-free mass; WC, Waist circumference; DBP, Diastolic blood pressure; SBP, Systolic blood pressure; MGS, Maximal grip strength; SU, Sit-up; SLJ, Standing long jump; CI, Confidence interval.

**Table 6:** Comparative analysis of body composition, blood pressure, and physical fitness in elderly women according to obesity level.

Variable	Under (n = 233)	Normal (n = 1958)	NWO (n = 1932)	Obesity (n = 9192)	*p*
FFM (kg)	32.18 (30.05–35.13)	37.07 ^ӨӨӨ###^ (34.76–39.26)	33.82 ^Ө^ (31.74–35.75)	37.47 ^ӨӨӨ^***^###^ (34.95–40.07)	0.001
WC (cm)	67.50 (64.05–71.15)	74.00 ^ӨӨӨ^ (70.90–78.00)	78.00 ^ӨӨӨ^*** (74.70–81.00)	86.00 ^ӨӨӨ^***^###^ (81.70–91.00)	0.001
DBP (mmHg)	70.00 (63.00–80.00)	74.00 (65.75–80.00)	75.00 ^ӨӨ^* (67.00–81.00)	76.00 ^ӨӨӨ^***^###^ (69.00–82.00)	0.001
SBP (mmHg)	129.00 (113.50–142.00)	129.00 (118.00–142.00)	132.00 ^ӨӨӨ^*** (120.00–145.00)	135.00 ^ӨӨӨ^***^###^ (124.00–146.00)	0.001
MGS (kg)	19.60 (16.45–22.83)	22.10 ^ӨӨӨ###^ (19.10–24.90)	20.70 (17.90–23.40)	21.80 ^ӨӨӨ###^ (18.80–24.90)	0.001
30 s CST (reps)	18.00 (13.00–22.00)	19.00 ^Ө###+++^ (15.00–23.00)	18.00 ^+++^ (14.00–22.00)	17.00 (14.00–21.00)	0.001
TUG (s)	6.19 (5.34–7.20)	5.87 (5.20–6.94)	6.01 ** (5.32–6.93)	6.26 ***^###^ (5.53–7.28)	0.001
F8WT (s)	25.50 (22.39–30.61)	24.50 ^ӨӨ^ (21.80–28.11)	25.03 *** (22.37–28.71)	26.00 ***^###^ (23.00–30.02)	0.001

Values are presented as the mean ± standard deviation. NWO, Normal weight obesity; FFM, Fat-free mass; WC, Waist circumference; DBP, Diastolic blood pressure; SBP, Systolic blood pressure; MGS, Maximal grip strength; 30 s CST, 30 s chair stand test; TUG, Timed up and go; F8WT; Figure-of-8 walk test; ^Ө^, *p* < 0.05 vs. Under; ^ӨӨ^, *p* < 0.01 vs. Under; ^ӨӨӨ^, *p* < 0.001 vs. Under; *, *p* < 0.05 vs. Normal; **, *p* < 0.01 vs. Normal; ***, *p* < 0.001 vs. Normal; ^###^ *p* < 0.001 vs. NWO; ^+++^ *p* < 0.001 vs. Obesity.

**Table 7:** Results of the odds ratio analysis of obesity regarding the body composition, blood pressure, and physical fitness of elderly women.

	Under		NWO		Obesity	
	OR (95% CI)	*p*	OR (95% CI)	*p*	OR (95% CI)	*p*
FFM (kg)	0.69 (0.67–0.72)	0.001	0.77 (0.76–0.79)	0.001	1.03 (1.02–1.04)	0.001
WC (cm)	0.85 (0.83–0.87)	0.001	1.11 (1.10–1.13)	0.001	1.38 (1.36–1.40)	0.001
DBP (mmHg)	0.98 (0.97–1.00)	0.008	1.01 (1.01–1.02)	0.004	1.03 (1.02–1.03)	0.001
SBP (mmHg)	0.99 (0.98–1.00)	0.020	1.01 (1.01–1.02)	0.001	1.02 (1.02–1.03)	0.001
MGS (kg)	0.90 (0.88–0.91)	0.001	0.94 (0.93–0.95)	0.001	0.99 (0.98–1.00)	0.081
30 s CST (reps)	0.97 (0.95–0.99)	0.008	0.98 (0.97–0.99)	0.001	0.96 (0.95–0.97)	0.001
TUG (s)	1.13 (1.07–1.20)	0.001	1.06 (1.03–1.10)	0.001	1.14 (1.11–1.17)	0.001
F8WT (s)	1.04 (1.02–1.05)	0.001	1.02 (1.01–1.03)	0.001	1.03 (1.03–1.04)	0.001

Values are presented as the mean ± standard deviation. NWO, normal weight obesity; FFM, Fat-free mass; WC, Waist circumference; DBP, Diastolic blood pressure; SBP, Systolic blood pressure; MGS, Maximal grip strength; 30 s CST, 30 second chair stand test; TUG, Timed up and go; F8WT, Figure-of-8 walk test.

## Data Availability

The data presented in this study are available upon request from the corresponding author.
